# Vitiligo-like depigmentation following neoadjuvant chemotherapy combined with pembrolizumab in early triple-negative breast cancer: a case report

**DOI:** 10.3389/fonc.2026.1817257

**Published:** 2026-07-20

**Authors:** Yinglei Deng, Dahua Mao, Shaogang Zhang, Jing Wang

**Affiliations:** Department of Breast Diseases, Affiliated Wudang Hospital of Guizhou Medical University, Guiyang, China

**Keywords:** immune-related adverse events, neoadjuvant immunotherapy, pembrolizumab, triple-negative breast cancer, vitiligo-like depigmentation

## Abstract

**Background:**

Triple-negative breast cancer (TNBC) is a biologically aggressive subtype of breast cancer associated with an unfavourable prognosis. In recent years, the combination of immune checkpoint inhibitors with neoadjuvant chemotherapy has become a standard treatment strategy for selected patients with early-stage TNBC. With the increasing use of immunotherapy, immune-related adverse events (irAEs) have received growing attention, among which cutaneous irAEs are the most frequently reported. However, vitiligo-like depigmentation remains a rare and infrequently described manifestation in the context of breast cancer immunotherapy.

**Case presentation:**

We report the case of a patient with early-stage triple-negative breast cancer who experienced tumour progression following anthracycline- and cyclophosphamide-based neoadjuvant chemotherapy at an outside institution. The patient was subsequently referred to our centre and treated with taxane- and carboplatin-based neoadjuvant chemotherapy in combination with pembrolizumab, resulting in a gradual reduction in tumour size on serial imaging assessments. Surgical resection was subsequently performed, with postoperative pathological evaluation confirming a pathological complete response. Around the fourth cycle of postoperative pembrolizumab maintenance therapy, the patient gradually developed vitiligo-like depigmentation predominantly involving the dorsal aspects of both hands and the perioral region, with additional scattered hypopigmented lesions on the trunk. These cutaneous changes were asymptomatic and did not interrupt the planned course of immunotherapy.

**Conclusion:**

This case suggests that vitiligo-like depigmentation may occur as a rare immune-related cutaneous manifestation in patients receiving pembrolizumab-based therapy for early-stage TNBC. Although this finding has no established predictive value for treatment outcomes in breast cancer, recognising this manifestation may help clinicians distinguish it from other dermatological conditions and avoid unnecessary treatment interruption when the lesions are mild and asymptomatic. Further reports are needed to clarify the incidence, biological basis, and clinical significance of immune-related depigmentation in patients with breast cancer receiving immune checkpoint inhibitors.

## Introduction

TNBC is a biologically aggressive subtype of breast cancer characterised by a high risk of recurrence. Owing to the absence of hormone receptor expression and human epidermal growth factor receptor 2 (HER2) amplification, therapeutic options for TNBC have historically been limited, and clinical outcomes have remained inferior to those of other molecular subtypes (1). In recent years, however, immunotherapy has led to significant advances in the treatment of early-stage TNBC (2). Clinical trials have demonstrated that the addition of pembrolizumab to neoadjuvant chemotherapy significantly increases pathological complete response rates compared with chemotherapy alone, as shown in the KEYNOTE-522 trial (3).

With the increasing use of immunotherapy in early-stage breast cancer, irAEs have received growing attention (4). Previous research has primarily focused on commonly encountered irAEs, including thyroid dysfunction, cutaneous reactions, and gastrointestinal toxicities (4, 5). In malignancies such as melanoma, vitiligo-like depigmentation is regarded as a distinctive immune-related cutaneous manifestation, and several studies have suggested a potential association between its occurrence and treatment response as well as survival outcomes (6, 7).

At present, the literature regarding the clinical significance of vitiligo-like depigmentation as an immune-related skin manifestation in breast cancer remains limited, particularly in patients receiving perioperative or postoperative immunotherapy (8). In this report, we describe a patient with early-stage TNBC who developed vitiligo-like depigmentation during pembrolizumab maintenance therapy after achieving a pathological complete response following neoadjuvant chemotherapy combined with pembrolizumab. We further discuss the potential clinical implications of this immune-related manifestation in the context of the existing literature.

## Case presentation

A 52-year-old female patient with a history of multiple prior excisions of benign breast lesions, including fibroadenoma and atypical hyperplasia, presented for further evaluation. She had not received any systemic therapy following these procedures and had been under regular follow-up. The patient denied a history of autoimmune disease or pigmentary skin disorders and reported no definite family history of malignancy.

The patient presented after noticing a palpable mass in the right breast. Physical examination revealed a firm mass measuring approximately 5.4 × 4.5 cm in the 10–12 o’clock position of the right breast, with relatively well-defined margins and mild local tenderness. No clinically enlarged lymph nodes were palpated in either axilla or the supraclavicular regions. In January 2023, a core needle biopsy of the right breast lesion was performed at an outside institution, and histopathological examination confirmed invasive breast carcinoma. Immunohistochemical analysis demonstrated negativity for oestrogen receptor (ER) and progesterone receptor (PR), absence of human epidermal growth factor receptor 2 (HER2) overexpression (score 0), and a Ki-67 proliferation index of approximately 70%, consistent with a diagnosis of triple-negative breast cancer. Concurrent axillary lymph node assessment showed no definite evidence of metastatic involvement. Germline BRCA1/2 testing did not identify pathogenic variants with established therapeutic relevance.

After completing four cycles of anthracycline- and cyclophosphamide-based neoadjuvant chemotherapy at an outside institution, reassessment demonstrated interval tumour enlargement on imaging, accompanied by increased firmness on palpation. The patient was subsequently referred to our centre for further management. In April 2023, repeat core needle biopsy of the breast lesion was performed at our institution, with histopathological findings and immunophenotype consistent with the previous diagnosis. Fine-needle aspiration of the right axillary lymph nodes revealed no definitive evidence of malignant cells, and the axilla was therefore considered clinically node-negative on overall assessment.

After contraindications to chemotherapy and immunotherapy had been excluded, the patient received weekly taxane- and carboplatin-based neoadjuvant therapy for 12 weeks, together with pembrolizumab 200 mg administered intravenously every 3 weeks. Imaging assessments during treatment showed gradual reduction in tumour size, consistent with an ongoing treatment response.

**Figure 1:**
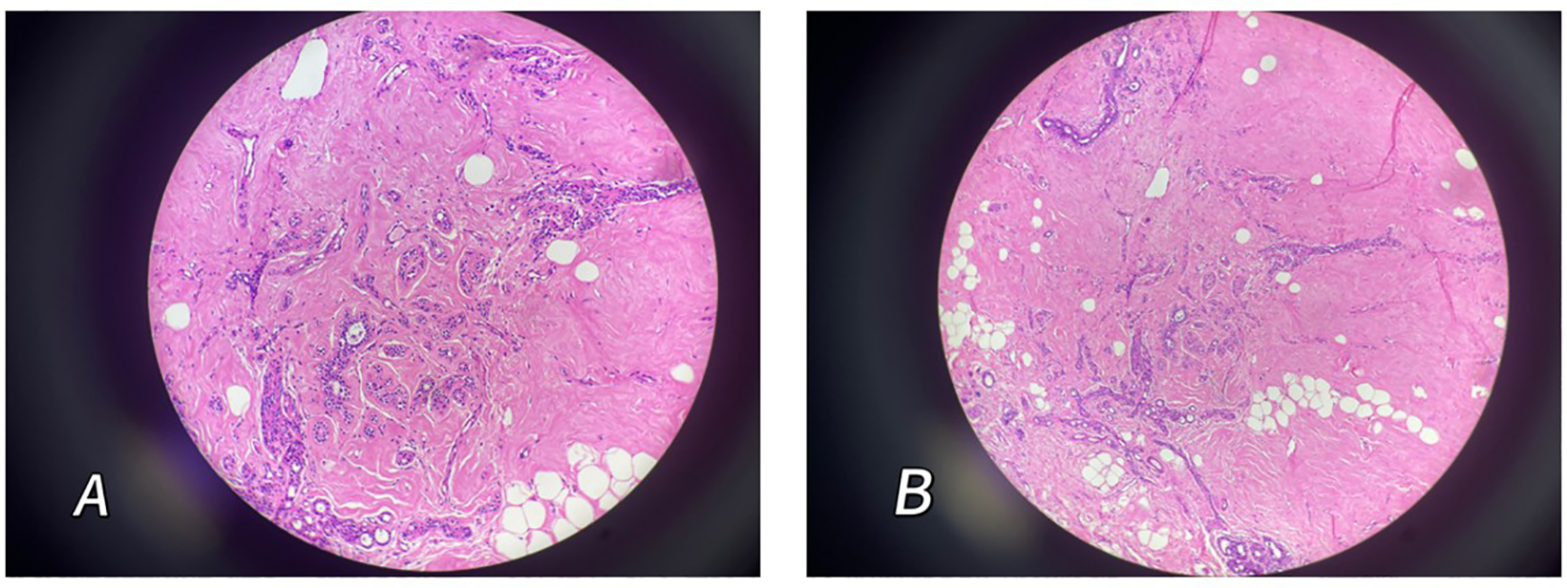
Histopathological findings of the breast surgical specimen after neoadjuvant immunotherapy. **(A)** Haematoxylin and eosin (H&E) staining of the breast surgical specimen at low magnification showing extensive fibrosis and treatment-related changes, with no definite residual invasive carcinoma identified. **(B)** H&E staining from another sampled area at low magnification demonstrating stromal fibrosis and residual ductal structures, without evidence of tumour cell persistence, consistent with a pathological complete response.

In September 2023, the patient underwent breast-conserving surgery of the right breast with sentinel lymph node biopsy. Postoperative pathological examination of the surgical specimen, following complete tissue sampling, revealed no definite residual invasive carcinoma. The sentinel lymph nodes and surrounding adipose tissue showed no evidence of metastatic disease, consistent with a pathological complete response after neoadjuvant therapy (**Figure 1**).

**Figure 2:**
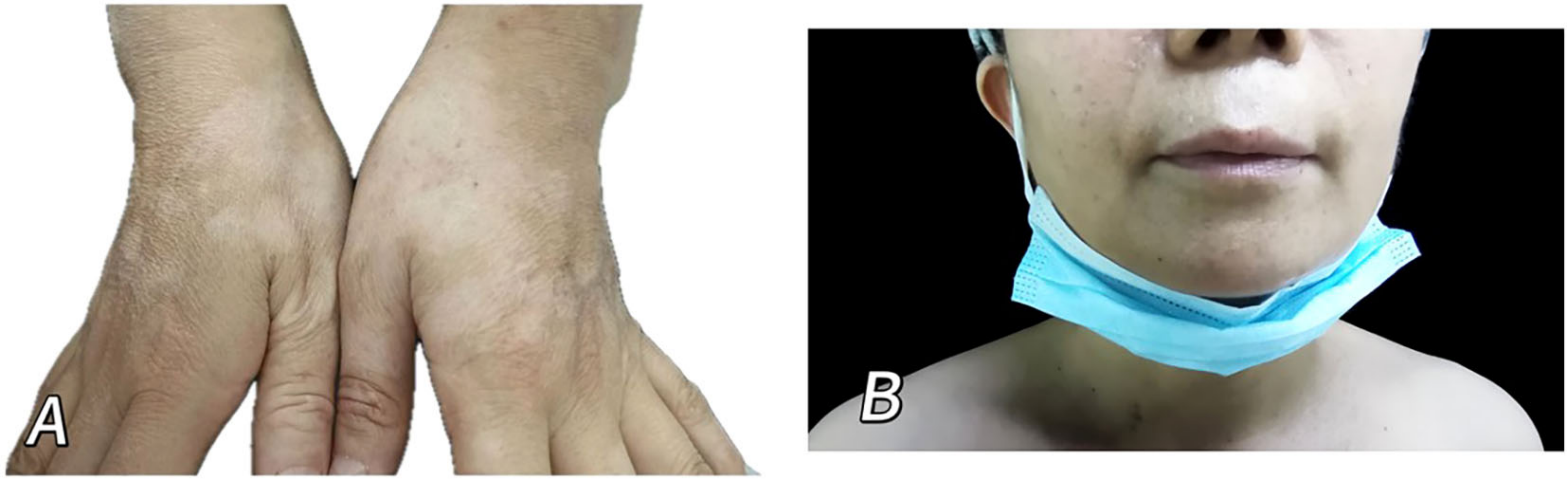
Vitiligo-like depigmentation during postoperative pembrolizumab maintenance therapy. **(A)** Bilateral hypopigmented patches involving the dorsal aspects of both hands. **(B)** Symmetrical depigmentation observed in the perioral region.

Postoperatively, the patient continued pembrolizumab as maintenance therapy to complete the planned one-year pembrolizumab course. Around the fourth cycle of postoperative pembrolizumab maintenance, approximately 8–9 weeks after initiation of maintenance treatment, she gradually developed hypopigmented skin changes predominantly involving the dorsal aspects of both hands and the perioral region. The lesions showed a bilateral distribution with an overall near-symmetrical appearance. Mild, scattered hypopigmented patches were also observed on the trunk. These cutaneous manifestations were not associated with pruritus, pain, erythema, scaling, or other inflammatory changes, and did not interfere with daily activities (**Figure 2**).

After noticing the depigmented lesions, the patient asked whether these skin changes could be related to her treatment. The clinical team explained that the findings were clinically compatible with an immune-related cutaneous manifestation, and that further dermatological assessment, including Wood lamp examination or skin biopsy, could be considered if diagnostic confirmation was considered necessary. However, as the lesions were mild, asymptomatic, and had no evident impact on daily activities, no additional investigations were pursued at that stage, particularly as the patient preferred not to undergo skin biopsy. After discussion, she expressed a preference to complete the planned pembrolizumab course if it remained clinically safe to do so, while agreeing that treatment interruption would be reconsidered if the depigmentation progressed or if additional immune-related toxicity developed. She remained adherent to follow-up and reported overall satisfaction with the treatment outcome.

**Figure 3:**
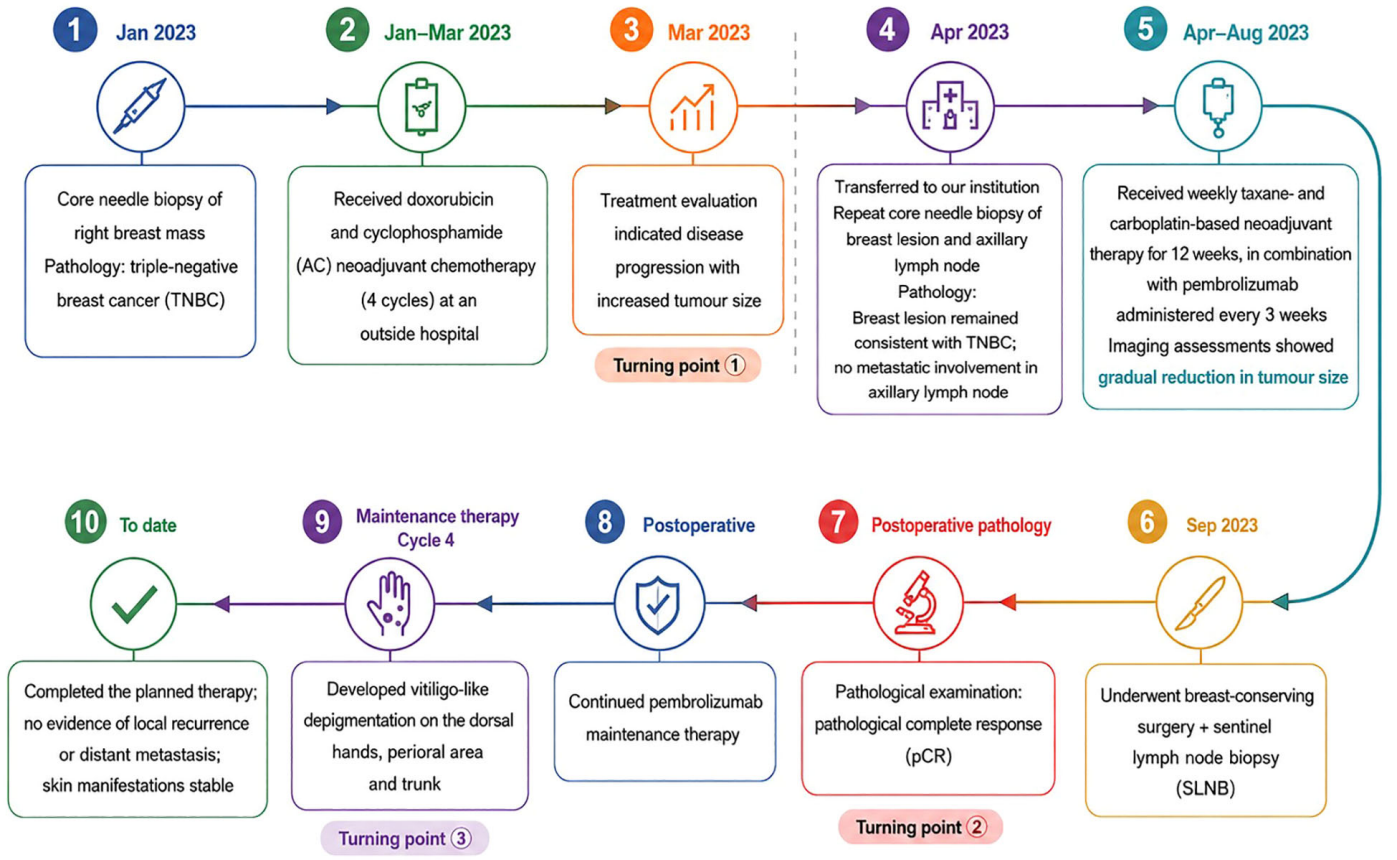
Timeline of the patient’s diagnostic and treatment course. The timeline summarises the clinical course from initial diagnosis of triple-negative breast cancer, progression after anthracycline- and cyclophosphamide-based neoadjuvant chemotherapy, subsequent taxane- and carboplatin-based chemotherapy combined with pembrolizumab, breast-conserving surgery with sentinel lymph node biopsy, pathological complete response, postoperative pembrolizumab maintenance therapy, onset of vitiligo-like depigmentation, and follow-up status.

The skin findings did not lead to interruption of immunotherapy, and the patient completed the planned one-year pembrolizumab course under close clinical follow-up. At a routine follow-up in April 2026, the patient remained clinically well, with no evidence of local recurrence or distant metastasis. The vitiligo-like depigmentation remained relatively stable during follow-up, without obvious progression or associated inflammatory symptoms, and did not require any specific dermatological treatment. She continues to undergo routine surveillance according to the institutional follow-up protocol. The overall clinical course of the patient is summarised in **Figure 3**.

## Discussion

In the present case, vitiligo-like depigmentation occurred during postoperative pembrolizumab maintenance therapy after a pathological complete response had been achieved. Although the temporal relationship is clinically noteworthy, current evidence remains insufficient to establish vitiligo-like depigmentation as a predictive marker of treatment response in breast cancer. Therefore, the value of this case lies not in suggesting a definitive association between depigmentation and therapeutic efficacy, but in documenting a visible and uncommon immune-related cutaneous manifestation in the setting of pembrolizumab-based treatment for early-stage TNBC.

An additional clinically relevant feature of this case is the change in treatment response after progression on anthracycline- and cyclophosphamide-based neoadjuvant chemotherapy. Despite interval tumour enlargement after four cycles of AC, the patient subsequently achieved a pathological complete response after taxane- and carboplatin-based chemotherapy combined with pembrolizumab. The early progression during AC may reflect the aggressive biology and high proliferative activity of TNBC, as well as potential intratumoural heterogeneity or early resistance to the initial chemotherapy regimen (1). This clinical course suggests that limited response to one neoadjuvant regimen does not necessarily preclude meaningful benefit from a subsequent platinum- and immunotherapy-containing approach. However, given the single-case nature of this report, the relative contributions of platinum-based chemotherapy and pembrolizumab to the final pathological response cannot be determined.

In this clinical context, the differential diagnosis of depigmented or hypopigmented lesions includes idiopathic vitiligo, post-inflammatory hypopigmentation, drug-induced pigmentary change, and other autoimmune or inflammatory skin disorders. In this patient, idiopathic vitiligo was considered less likely because she had no previous history of vitiligo, pigmentary disorder, or autoimmune disease. Post-inflammatory hypopigmentation was also considered less likely, as the lesions were not preceded or accompanied by erythema, scaling, pruritus, pain, or other inflammatory changes. Given the timing of onset during pembrolizumab maintenance therapy, the bilateral distribution involving the dorsal hands and perioral region, and the absence of alternative clinical explanations, the findings were considered most consistent with pembrolizumab-associated vitiligo-like depigmentation.

In breast cancer, the true incidence and clinical significance of vitiligo-like depigmentation during immune checkpoint inhibitor therapy remain unclear. In KEYNOTE-522, dermatologic adverse events were reported mainly as rash, pruritus, or immune-mediated rash/dermatitis, whereas vitiligo-like depigmentation was not separately characterized as a common pigmentary adverse event (3, 9). Similarly, real-world studies of pembrolizumab-based neoadjuvant therapy in early-stage TNBC have mainly focused on overall immune-related adverse events, endocrine toxicities, treatment discontinuation, and associations with pathological complete response (10, 11). Pigmentary adverse events have not been consistently reported as a separate category. Therefore, it remains uncertain whether vitiligo-like depigmentation is truly rare in patients with breast cancer or simply underreported, and its clinical significance in this setting remains unclear.

Immune-related adverse events associated with immune checkpoint inhibitors vary across tumour types, and cutaneous toxicities are among the most frequently reported irAEs (4, 8). Vitiligo-like depigmentation has been most extensively described in melanoma, where it is thought to reflect T-cell–mediated immune responses against melanocyte-associated antigens and has been associated with favourable treatment outcomes (6, 7, 12). However, these observations should be interpreted with caution in the breast cancer setting, as breast cancer does not arise from melanocytic lineage and has a distinct immunobiological context.

One possible explanation for immune checkpoint inhibitor-associated depigmentation is that enhanced T-cell activity may not be confined to tumour tissue, but may also involve immune recognition of antigens expressed in normal tissues. In selected patients, processes such as epitope spreading, bystander immune activation, or T-cell cross-reactivity may contribute to immune recognition of pigmentation-related antigens in the skin, thereby resulting in depigmentation (13). However, direct mechanistic evidence remains limited, particularly in breast cancer, and the present case cannot establish whether the observed skin changes were mechanistically linked to the pathological complete response. Therefore, vitiligo-like depigmentation in this case should be interpreted as an uncommon immune-related cutaneous manifestation rather than a validated biomarker of treatment efficacy.

Vitiligo-like depigmentation remains insufficiently characterized in the setting of breast cancer immunotherapy. Given the broader uncertainty regarding the relationship between immune-related adverse events and antitumour efficacy (14), further reports and larger studies are needed to clarify whether similar pigmentary changes have any consistent biological or clinical significance in patients with breast cancer receiving immune checkpoint inhibitors.

## Clinical implications

In the present case, continuation of pembrolizumab was considered reasonable because the depigmentation was mild, asymptomatic, not associated with inflammatory symptoms or systemic immune-related toxicity, and did not interfere with daily activities. The patient also wished to complete the planned pembrolizumab course if it remained safe to do so. Close clinical monitoring was maintained, with the understanding that treatment interruption would be reconsidered if the lesions progressed or if additional immune-related adverse events developed. The subsequent stability of the skin findings during follow-up supported the decision to complete the planned course of treatment.

## Limitations

A limitation of this report is that Wood lamp examination and skin biopsy were not performed. The diagnosis of vitiligo-like depigmentation was therefore based on the clinical appearance of the lesions and their temporal association with pembrolizumab treatment. In addition, as this is a single case report, no conclusion can be drawn regarding the predictive value of vitiligo-like depigmentation in patients with breast cancer receiving immune checkpoint inhibitors.

## Conclusion

We report a case of early-stage TNBC in which vitiligo-like depigmentation developed during postoperative pembrolizumab maintenance therapy after a pathological complete response had been achieved following neoadjuvant chemotherapy combined with pembrolizumab. Such manifestations remain rarely reported in the context of breast cancer immunotherapy and currently have no established role as predictors of treatment efficacy. Nevertheless, this case highlights a visible cutaneous immune-related manifestation during pembrolizumab treatment and underscores the importance of recognising and documenting uncommon skin findings in patients receiving immune checkpoint inhibitors.

## Data Availability

The original contributions presented in the study are included in the article/supplementary material. Further inquiries can be directed to the corresponding author.
